# Design of a new nanocomposite based on Keggin-type [ZnW_12_O_40_]^6−^ anionic cluster anchored on NiZn_2_O_4_ ceramics as a promising material towards the electrocatalytic hydrogen storage

**DOI:** 10.1038/s41598-024-61871-0

**Published:** 2024-05-14

**Authors:** Mohammad Ali Rezvani, Hadi Hassani Ardeshiri, Alireza Gholami, Masomeh Aghmasheh, Amir Doustgani

**Affiliations:** 1 Department of Chemistry, Faculty of Science, University of Zanjan, Zanjan, 451561319 Iran; 2 Catalysts and Organic Synthesis Research Laboratory, Department of Chemistry, Iran University of Science and Technology, Tehran, 16846-13114 Iran; 3 Department of Chemical Engineering, Faculty of Engineering, University of Zanjan, Zanjan, Iran

**Keywords:** Keggin-type polyoxometalate, Assembled nanocomposite, Hydrogen storage, NiZn_2_O_4_ nanoceramics, Catalysis, Inorganic chemistry, Environmental sciences, Chemistry

## Abstract

Extensive research efforts have been dedicated to developing electrode materials with high capacity to address the increasing complexities arising from the energy crisis. Herein, a new nanocomposite was synthesized via the sol–gel method by immobilizing K_6_ZnW_12_O_40_ within the surface of NiZn_2_O_4_. ZnW_12_O_40_@NiZn_2_O_4_ was characterized by FT-IR, UV–Vis, XRD, SEM, EDX, BET, and TGA-DTG methods. The electrochemical characteristics of the materials were examined using cyclic voltammogram (CV) and charge–discharge chronopotentiometry (CHP) techniques. Multiple factors affecting the hydrogen storage capacity, including current density (j), surface area of the copper foam, and the consequences of repeated cycles of hydrogen adsorption–desorption were evaluated. The initial cycle led to an impressive hydrogen discharge capability of 340 mAh/g, which subsequently increased to 900 mAh/g after 20 cycles with a current density of 2 mA in 6.0 M KOH medium. The surface area and the electrocatalytic characteristics of the nanoparticles contribute to facilitate the formation of electrons and provide good diffusion channels for the movement of electrolyte ions throughout the charge–discharge procedure.

## Introduction

While hydrogen is considered as a highly promising alternative fuel for energy production and consumption systems due to its clean-burning properties, its relatively low volumetric energy density has hindered its sorption abilities under ambient conditions. Thus, the consideration of hydrogen storage is crucial for the widespread utilization of hydrogen due to its lower costs, less environmental damages, and the availability of the primary sources^1–3^. The combustion of hydrogen results in the production of water as a byproduct, thereby bestowing numerous environmental benefits upon it^4,5^. To address the demand for the advancement of sustainable energy, the focal point is directed towards the storage systems of clean energy, specifically hydrogen^6,7^. A significant amount of effort has been dedicated to the exploration of new nanomaterials that possess the ability to enhance the capacity for hydrogen storage across various operational conditions. The storage of hydrogen is achieved through three distinct methods, which comprise the storage of compressed gas within containers, the storage of hydrogen in the form of liquid or solid^8^. Due to the necessity for high pressure and the construction of sizable containers, the viability of compressed hydrogen storage is limited from an economic standpoint. Factors such as the substantial expense associated with the construction of a large hydrogen storage container, the requirement for ample space to accommodate the hydrogen storage containers, the considerable weight of the containers, and the elevated pressure required for hydrogen liquefaction contribute to this limitation^9,10^. The storage of hydrogen in liquid form, also known as cryogenic storage, necessitates a reduction in temperature to 20 °C. To ensure temperatures remain below freezing point, a hydrogen storage container must be adequately insulated^11^. Despite recent advancements in liquefaction techniques, this approach is not widely employed today due to its exorbitant cost. Conversely, solid-state hydrogen storage presents numerous advantages, encompassing enhanced safety measures, a heightened capacity for voluminous storage, as well as lower operating temperatures and pressures. Among the different methods and limitations mentioned, electrochemical hydrogen storage emerges as the most effective method amidst various approaches, due to its ability to produce and retain hydrogen in-situ, while functioning under normal pressure and temperature conditions^12^. Polyoxometalates (POMs) have recently attracted considerable interest in the field of hydrogen storage due to their notable chemical and structural variability, redox potential, surface charge, thermal stability, and adjustable acidity^13,14^. POMs are a class of inorganic compounds that consist of metal-oxo clusters, typically containing a combination of transition metals and oxygen atoms. Some common structures of POMs, e.g., Keggin [{XM_12_O_40_}^3/4−^] (X = P, Si, B and M = and M = Mo, W)^15^, sandwich [{M_4_(H_2_O)_2_(XW_9_O_34_)_2_}^n−^ (X = P, Si, Ga, etc.)^16^, Anderson [{AM_6_O_24_}^9/10−^] (A = Mn, Fe, Cr, Al, I, etc. and M = Mo, W)^17^, Wells–Dawson [{X_2_M_18_O_62_}^6−^], (X = P, Si and M = Mo, W)^18^, Evans–Showell [{Co_2_Mo_10_H_4_O_38_}^6^]^19^, etc. Among their various configurations, Keggin-type POMs have been designed for the purpose of catalyzing the hydrogen storage systems. This is made possible by their possession of multiple redox-active metal centers and bridging oxygen atoms, which enable them to accept and denote electrons during the reaction^20,21^. Nevertheless, there are certain drawbacks associated with utilization of POMs in industrial applications, including a limited surface area (5 m^2^ g^−1^) and poor reusability due to its high solubility^22,23^. Hence, the immobilization of Keggin-type POMs on the surfaces of metal oxides and polymers emerges as a promising approach to enhance their catalytic properties and mechanical durability. To design an innovative heterogeneous catalyst that possesses high stability and impressive reusability, a range of materials based on metallic oxides were employed as careers for the support of POMs, e.g., MgCo_2_O_4_,NiZn_2_O_4_,CuCo_2_O_4_, NiCo_2_O_4_, etc^24,25^. The NiZn_2_O_4_ nanoparticles have become a preferred material for use in the heterogenization of POMs due to their non-toxic nature, low cost of fabrication, eco-friendly properties, and the specific capacities that arise from the synergistic impact of Ni and Zn ions^26^. In our research, we endeavor to investigate novel characteristics of Keggin-type POMs by substituting different metals. The Keggin structure consists of a spherical conglomerate of polymeric units of MO_6_ (M = Mo, W), wherein the corners and edges are shared, along with a central tetrahedron (PO_4_). Furthermore, POMs typically exhibit a weakly basic and nucleophilic nature, unless the surface charge is enhanced through metal substitution or by reducing the POMs. This research has validated that the incorporation of Zn metal into the tetrahedral unit of Keggin-type polyoxometalate leads to a substantial enhancement of the hydrogen storage. Herein, a new and efficient nanocomposite was synthesized based on Keggin-type [ZnW_12_O_40_]^6−^ and NiZn_2_O_4_ with hydrogen storage capability. The physicochemical characteristics of the as-prepared nanocomposite was characterized through several analyses. Subsequently, the hydrogen storage capacity was examined via CV and CHP techniques, exploring various factors. This study presents a potential method for hydrogen storage using the ZnW_12_O_40_/NiZn_2_O_4_ nanocomposite.

## Experimental section

### Materials

All chemicals and solvents used in this investigation were procured from commercial sources and utilized in accordance with the specified instructions. Zinc nitrate hexahydrate (Zn(NO_3_)_2_·6H_2_O, $\ge 98\%)$, nickel(II) nitrate hexahydrate (Ni(NO_3_)_2_·6H_2_O, $97\mathrm{\%})$, sodium tungstate dihydrate (Na_2_WO_4_·2H_2_O, $99\%$), phosphate Buffer Saline (PBS) were purchased from Sigma-Aldrich. Moreover, potassium chloride (KCl, ≥ 99%), citric acid monohydrate (HOC(COOH)(CH_2_COOH)_2_·H_2_O), urea (CH_4_N_2_O), ethanol (EtOH, 96%), and potassium hydroxide (KOH, ≥ 98%) were provided from Merck chemical company.

### Characterizations methods

The Fourier transform infrared (FT-IR) spectra were acquired using the Thermo-Nicolet-iS 10 spectrometer in the solid state from 400 to 4000 cm^−1^. The investigation of the optical characteristics was conducted through the utilization of a Shimadzu UV–Vis double beam spectrometer, specifically the UV-2450 model originating from Japan. This analysis was performed within the spectral range spanning from 200 to 700 nm. The measurement of X-ray diffraction (XRD) was executed by Bruker D8 Advance, which was outfitted with a crystal monochromator made of graphite. The XRD employed a voltage of 40 kV and a current of 30 mA, utilizing CuKα radiation with a wavelength of 0.15406 nm. The field-emission scanning electron microscope (FE-SEM) by LEO 1455 VP (voltage of 10.00 kV) was employed to examine the particle size and surface morphologies of the samples, along with an energy-dispersive X-ray spectroscopy (EDX) for elemental mappings. In addition, a simultaneous analysis of thermogravimetry (TGA) and derivative thermal gravimetry (DTG) was conducted on a NETZSCH STA 409 PC/PG Germany spectrometer. Additionally, the measurement of the specific surface area was carried out by BET device, specifically the Belsorp-Mini II, manufactured by Microtrace. The electrochemical analyses involving cyclic voltammetry (CV) and chronoamperometry (CHP) were performed using a Sama 500 potentiostat (Isfahan, Iran).

### Preparation of the ZnW_12_O_40_@NiZn_2_O_4_ nanocomposite

#### Synthesis of Keggin-type [ZnW_12_O_40_]^6–^ clusters

0.12 g (2.5 mmol) of Zn(NO_3_)_2_·6H_2_O and 1.38 g (25 mmol) of Na_2_WO_4_·2H_2_O were completely dissolved in 15 mL of DW, separately. Afterward, the prepared solution of Zn(NO_3_)_2_·6H_2_O was added gradually to Na_2_WO_4_·2H_2_O solution. Following this, the pH value was adjusted at 5.5 using buffer of acetate (1 M). The obtained solution was refluxed for 6 h and after cooling to room temperature, a saturated solution prepared from KCl (5.00 g in 10 mL of DW) was added and stirred for 60 min. Following a few days of aging, the resulting solid precipitate was washed using ethanol and water, and then was dried at 80 °C for 4 h.

#### Synthesis of NiZn_2_O_4_ nanoparticles

In order to synthesize NiZn_2_O_4_ ceramics^27^, the subsequent procedures were performed: Initially, Ni(NO_3_)_2_·6H_2_O (0.30 g) was dissolved in 20 mL of DW as a Ni precursor. Subsequently, this solution was added drop-wise to the Zn(NO_3_)_2_.6H_2_O solution (0.12 g in 20 mL of DW) with a molar ratio of 1:2 under magnetic stirring (referred to as solution A). Additionally, a mixture of citric acid solution (0.50 g in 15 mL of DW) and urea (0.32 g in 20 mL of DW) was prepared with a molar ratio of 1:2, employing sonication (referred to as solution B). The obtained solution was then heated to the temperature of 75 °C. Afterward, the combination of solutions A and B was carried out under constant agitation for 5 h at 80 °C, leading to the formation of a green gel. The resulting gel was subjected to calcination at a temperature of 500 °C for 4 h in a furnace, resulting in the generation of NiZn_2_O_4_ nanoparticles powder.

#### Synthesis of ZnW_12_O_40_/NiZn_2_O_4_ nanocomposite

In order to prepare the ZnW_12_O_40_/NiZn_2_O_4_ nanocomposite, the following method was performed via the sol–gel method. In a typical procedure, the gradual addition of Keggin-type ZnW_12_O_40_ solution (0.09 g in 20 mL of DW) to the prepared gel consisting of NiZn_2_O_4_ nanoparticles (prior to complete gelling) was conducted. Subsequently, the resulting solution was subjected to magnetic agitation at 80 °C for 60 min, leading to the formation of a gray gel. Following this, the gray gel that was acquired was subjected to calcination process in a furnace, wherein it was exposed to a temperature of 500 °C for 4 h, resulting in the formation of the ZnW_12_O_40_/NiZn_2_O_4_ powder.

### Electrochemical hydrogen storage study

**Scheme 1.**
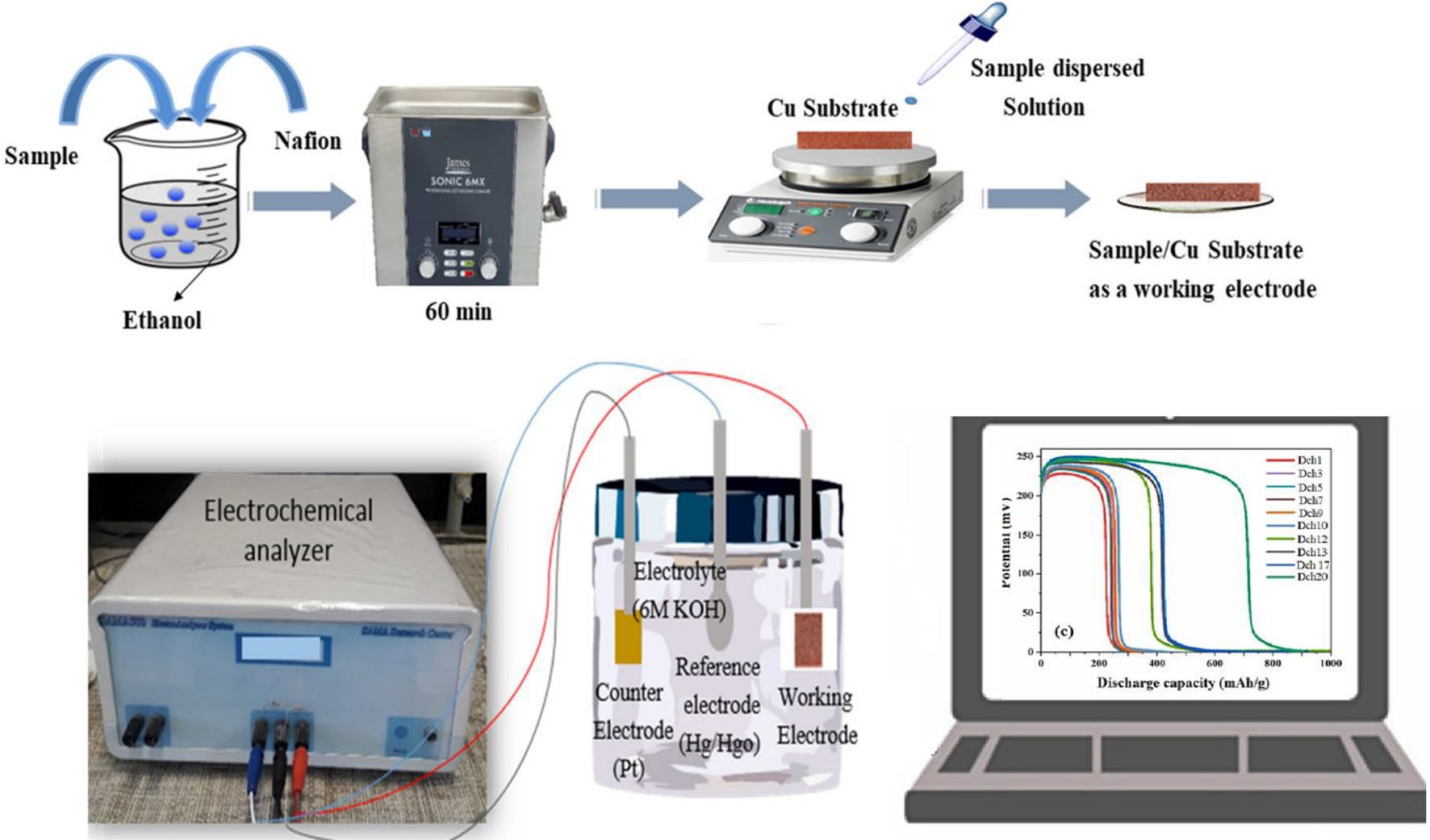
Schematic illustration of working electrode (sample/Cu substrate) and a view of the electrochemical cell.

The CHP technique was employed to determine the hydrogen storage capacity of the ZnW_12_O_40_/NiZn_2_O_4_ nanocomposite electrodes in a three-electrode electrochemical cell comprising a reference electrode (Hg/HgO electrode), a counter electrode (Pt plate), and a working electrode (bare and modified copper foam). For the purpose of preparing the electrolyte solution, the aqueous electrolyte with a molarity of 6.0 M KOH was provided. To prepare 6 M KOH, 16.83 g of solid KOH was dissolved in distilled water and then added to the desired volume in a 50 mL of volumetric flask. A working electrode was created by coating a bare copper plate ((porous per inch) PPI: 95, 1 $\times$ 2 cm^2^) with a thin layer of the ZnW_12_O_40_/NiZn_2_O_4_ nanocomposite powders at a temperature of 100 °C for 60 min, without the use of any binder. The working electrode was prepared in the following manner: the synthesized samples were dispersed in EtOH using an ultrasonic bath. Subsequently, the dispersion was applied onto the Cu foam through the drop cast method, and coated Cu foam was dried in an oven for 10 h. The electrochemical cell was assembled and operated at room temperature. By applying a constant current between the working and counter electrodes, the potential of the ZnW_12_O_40_@NiZn_2_O_4_ nanocomposite electrode was measured in relation to the reference electrode. In the following, the charge–discharge galvanostatic technique was executed on the SAMA-500 device at the designated current density of 2 mA. Moreover, The CV analysis for the achieved electrodes was carried out in analogous assembled cells at a scan rate of 0.10 Vs^−1^. Scheme 1 illustrates a diagrammatic representation of the procedure for preparing the working electrode and a view of the electrochemical cell.

## Results and discussion

### Characterization of ZnW_12_O_40_@NiZn_2_O_4_ nanocomposite

#### FT-IR spectra

**Figure 1:**
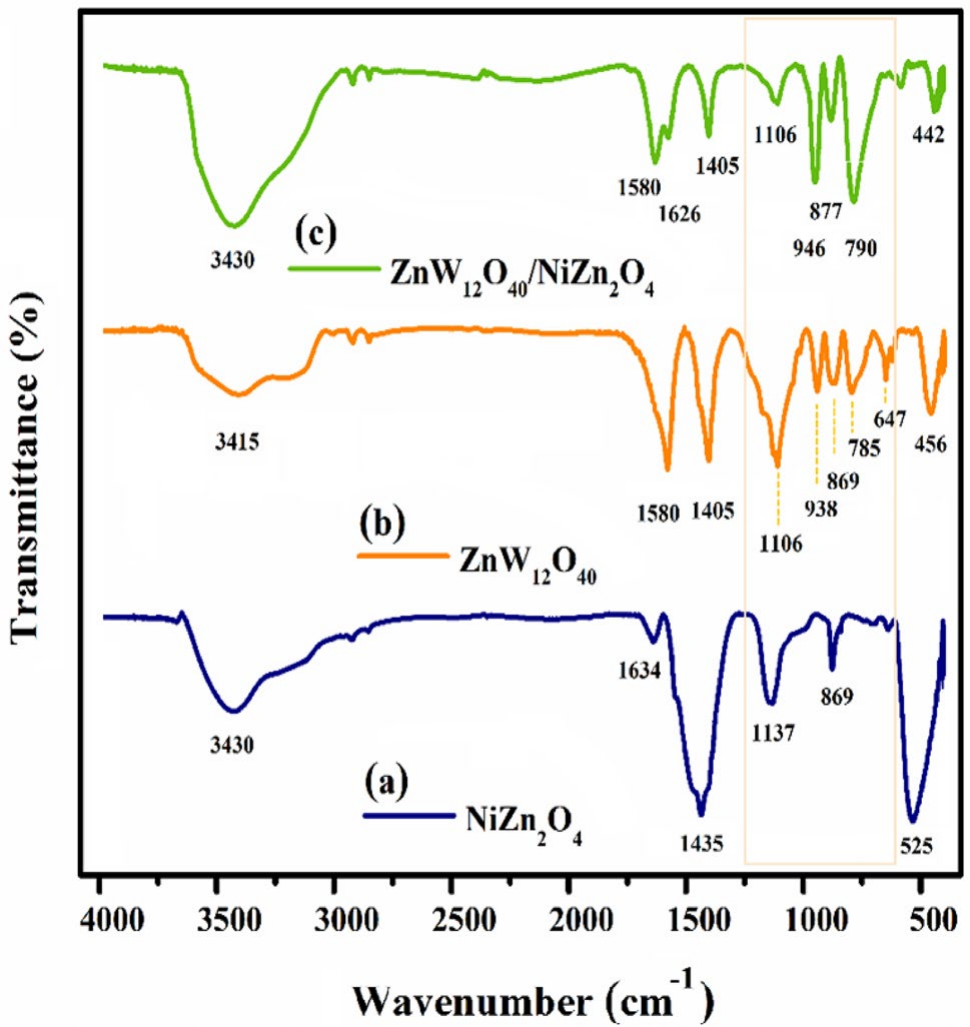
FT-IR spectra of (**a**) NiZn_2_O_4_, (**b**) ZnW_12_O_4_, and (**c**) ZnW_12_O_40_/NiZn_2_O_4_ nanocomposite.

The successful preparation of the ZnW_12_O_40_@NiZn_2_O_4_ nanocomposite was accomplished through the FT-IR spectroscopic analysis. The sequence FT-IR spectra of (a) NiZn_2_O_4_, (b) ZnW_12_O_4_, and (c) ZnW_12_O_40_/NiZn_2_O_4_ nanocomposite can be found in Fig. 1 with a spectral range of 400–4000 cm^−1^. As pointed out in Fig. 1a, the characteristic peaks at 525 and 1137 cm^−1^ are attributed to the stretching vibrations of Zn–O and Ni–O–Zn groups, respectively^28^. Additionally, the characteristic peak at 869 cm^−1^ is assigned to the stretching vibration band of Ni–O–H functional group. Furthermore, the absorption bands positioned at 3430 and 1634 cm^−1^ are associated with the bending and stretching vibrations of H–O–H molecules, respectively. It is worth noting that the absorption of air moisture during the preparation of sample pellets is the cause of these phenomena^29^. These observations have thus provided valuable evidence concerning the influence of hydration on the structure of matter. The corresponding vibrational frequencies of the prepared [ZnW_12_O_40_]^6–^ anionic clusters are observed at 1106, 938, 869, and 785 cm^−1^, which are related to the stretching vibrations of Zn-Oa (central bond), W = Od (terminal bond), W–O_b_–W (corner-sharing bond), and W–Oc–W (edge-sharing bond), respectively (Fig. 1b)^30,31^. As illustrated in Fig. 1c, peaks associated with the presence of [ZnW_12_O_40_]^6–^ were observed in the resulting as-prepared nanocomposite, exhibiting a certain degree of displacement. Furthermore, it was observed that the characteristic [ZnW_12_O_40_]^6–^ peaks displayed a partial coverage by the ZnW_12_O_40_/NiZn_2_O_4_ bands at 1348, 1454, and 482 cm^−1^. The findings provided an initial validation for the nanocomposite of [ZnW_12_O_40_]^6–^ supported on NiZn_2_O_4_ ceramics solid. As a result, the successful immobilization of Keggin-type POM on NiZn_2_O_4_ matrix can be effectively demonstrated via the FT-IR surveys.

#### UV–Vis analysis

**Figure 2:**
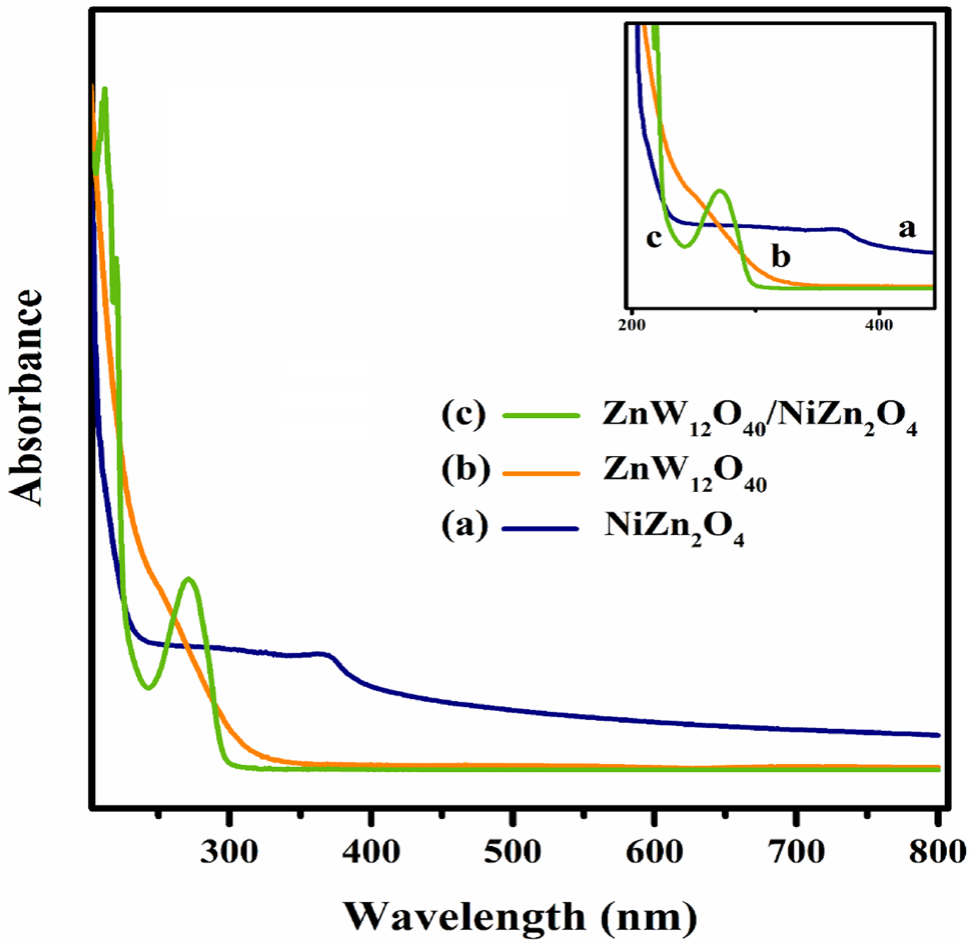
UV–Vis spectra of (**a**) NiZn_2_O_4_, (**b**) ZnW_12_O_4_, and (**c**) ZnW_12_O_40_/NiZn_2_O_4_.

In order to investigate the charge transfer mode of the materials, the UV–Vis spectra of the (a) NiZn_2_O_4_, (b) ZnW_12_O_4_, and (c) ZnW_12_O_40_/NiZn_2_O_4_ nanocomposite were displayed in Fig. 2. As illustrated in Fig. 2a, the absorption peaks in the region of 300–400 nm are possibly caused due to the ligand to metal charge transfer (LMCT) of oxygen (2p) to metal species (O^2−^ → Zn^2+^ and/or O^2−^ → Ni^2+^) and π → π* transitions of metals. According to the spectrum of Keggin-type [ZnW_12_O_40_]^6–^ (Fig. 2b), absorption peaks at 257 and 315 nm can be attributed to the LMCT of tetrahedral oxygen (2p) to tungsten (O^2−^_(2p)_ → W^6+^) and bridge oxygens (2p) to tungsten (O^2−^_b/c_ → W^6+^), respectively^32,33^. Furthermore, the shifts towards shorter hypochromic wavelengths (blue-shifts) can be seen compared to the pure components of as-prepared nanocomposite material (Fig. 2c).

#### Bandgap determination

**Figure 3:**
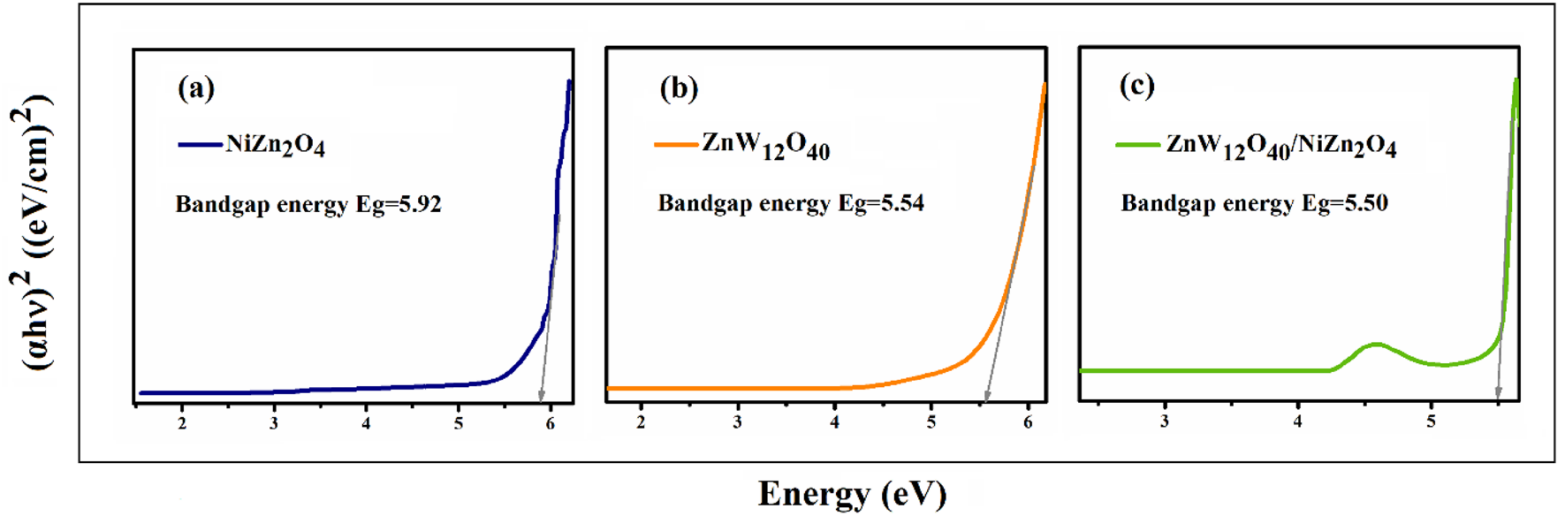
Band gap energies (Eg) of (**a**) NiZn_2_O_4_, (**b**) ZnW_12_O_4_, and (**c**) ZnW_12_O_40_/NiZn_2_O_4_ using experimental data of UV–Vis.

The estimation of the bandgap energies of the materials has been conducted using the classical Tauc relation, in adherence to the subsequent methodology^34^:

1 $$ \mathop {{(}\alpha {\text{h}}\upsilon )}\nolimits^{\frac{1}{n}} = A(h\upsilon - Eg) $$

where A represents a constant known as the band tailing parameter, $\alpha$ denotes the absorption coefficient, Eg refers to the energy of the optical band gap, h represents the Planck constant, v signifies the frequency of the photon, and n represents the power factor of the transition mode^35^. The curves of (αhv)^2^ versus hv for (a) NiZn_2_O_4_, (b) ZnW_12_O_4_, and (c) ZnW_12_O_40_/NiZn_2_O_4_ nanocomposite are pointed out in Fig. 3. The relationship between (αhv)^2^ and hυ (energy) exhibits a linear pattern within a specific area. Extending this linear pattern will result in the intersection of the (hυ)-axis, providing the determination of the optical energy gap (Eg). Based on the calculations conducted, the bandgap energies of NiZn_2_O_4_, Keggin-type [ZnW_12_O_40_]^6^, and ZnW_12_O_40_/NiZn_2_O_4_ nanocomposite were determined to be 5.92, 5.54, and 5.50, correspondingly. Lower band gap of as-prepared nanocomposite typically exhibits higher carrier densities compared to materials with larger band gaps. This is because a smaller band gap allows more electrons to be excited from the valence to the conduction band, resulting in a higher concentration of free charge carriers.

#### XRD data

**Figure 4:**
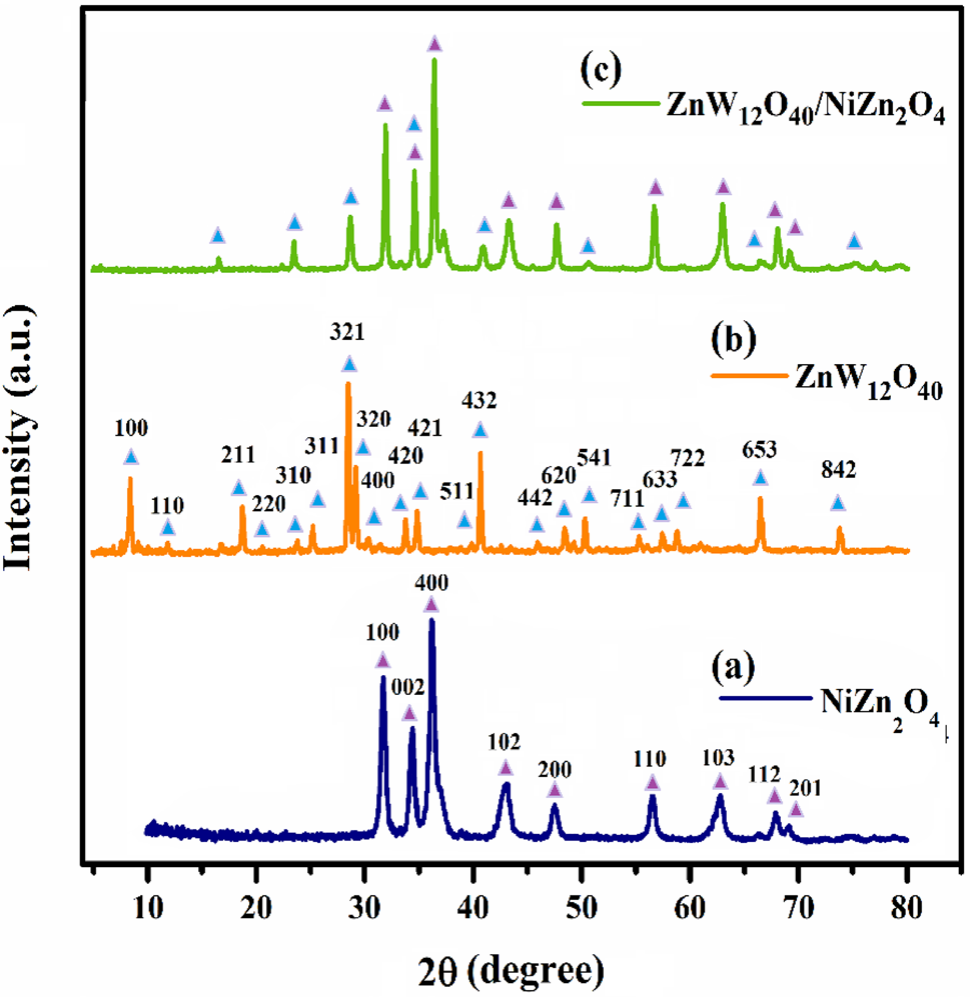
XRD patterns of (**a**) NiZn_2_O_4_, (**b**) ZnW_12_O_4_, and (**c**) ZnW_12_O_40_/NiZn_2_O_4_ nanocomposite.

The investigation of the XRD pattern of materials was conducted in order to examine the phase and dimensions of both grains and nanoparticles based on Rayleigh scattering (Fig. 4). According to the data presented in Fig. 4a, the prominent diffraction peaks observed at 2θ = 31.5, 34.3, 36.1, 43.1, 47.7, 56.8, 62.9, 68.1, and 69.0° can be attributed to the crystallographic planes denoted as (100), (002), (400), (102), (200), (110), (103), (112), and (201) within the hexagonal phase of NiZn_2_O_4_^36,37^. Furthermore, there are prominent diffraction peaks observed at angles of 2θ = 8.3°, 11.8°, 18.8°, 20.5°, 23.8°, 2.26°, 28.5°, 30.3°, 33.8°, 34.8°, 36.9°, 40.6°, 41.2°, 45.9°, 48.4°, 50.3°, 57.4°, 58.7°, 60.0°, 66.4°, and 73.7°, which correspond to the crystal planes of (100), (110), (211), (220), (310), (311), (321), (320), (400), (420), (421), (511), (432), (442), (620), (541), (711), (633), (722), (653), and (842) (Fig. 4b)^38^. These peaks are indicative of the presence of potassium zinc tungsten oxide and suggest the existence of Keggin-type [ZnW_12_O_40_]^6−^ species, as confirmed by the JCDD card No. 00-043-0001. The diffraction pattern of the ZnW_12_O_40_/NiZn_2_O_4_ nanocomposite reveals the presence of the majority of characteristic peaks associated with ZnW_12_O_40_-heteropolyoxo and NiZn_2_O_4_ within the structure of the nanocomposite, albeit with a slight shift (Fig. 4c). Upon formation of the composite, there exists the potential for alterations in individual crystal planes and potential variations in grain population orientations that result in crystal planes exhibiting specific Miller index orientations. Additionally, it is plausible that the peaks of the pure amorphous background material may coincide with similar peaks. The displacement and disappearance of certain [ZnW_12_O_40_]^6–^ peaks can primarily be attributed to the rearrangement of Keggin clusters anions during the self-assembly process of [ZnW_12_O_40_]^6–^ species and ceramic ions. Furthermore, the variation in atomic radius between the NiZn_2_O_4_ nanoparticles and the [ZnW_12_O_40_]^6–^ serves as another factor leading to the reduction in intensity and shift of multiple peaks. Ultimately, the utilization of the Debye-Scherer formula enables the determination of the average crystallite size of the ZnW_12_O_40_/NiZn_2_O_4_ nanocomposite, which is calculated to be approximately 32.9 nm^39,40^. Besides, the X-ray peak broadening analysis was implemented to investigate the dimensions of the crystals and the lattice strain through the Williamson–Hall (W–H) in the subsequent manner^41,42^:

2 $$ \beta_{hkl} {\text{cos}}\theta { = }\frac{k\lambda }{D} \, + 4\varepsilon {\text{sin}}\theta $$

**Figure 5:**
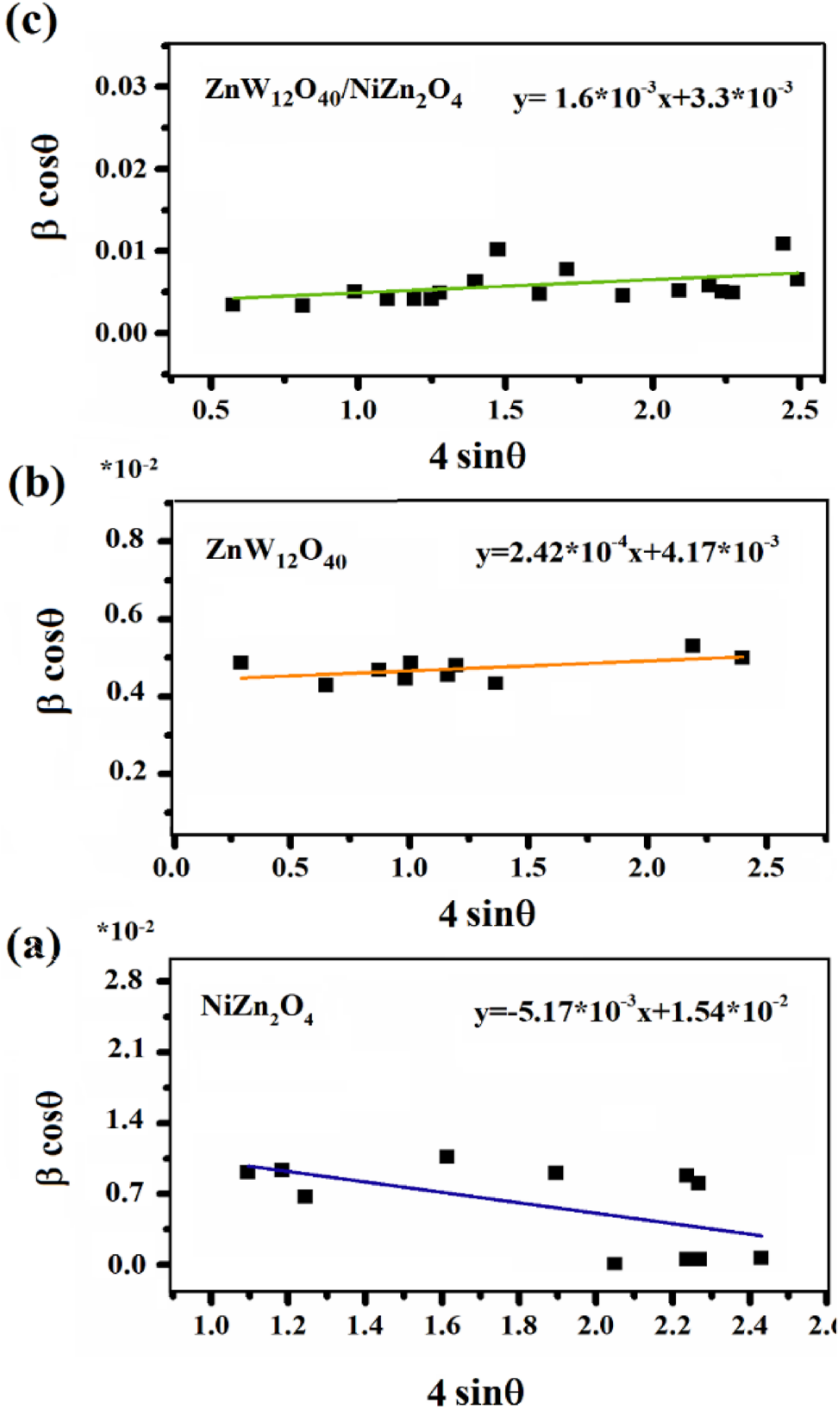
W–H plots of (**a**) NiZn_2_O_4_, (**b**) ZnW_12_O_4_, and (**c**) ZnW_12_O_40_/NiZn_2_O_4_ nanocomposite.

In this formula, β_hkl_ corresponds to the broadening of the instrumental peak, D represents the size of the crystallites (nm), K is the factor of shape which is equivalent to 0.89, λ corresponds to the wavelength of X-rays (0.15406 nm), θ denotes the reflection angle, and ε stands for the strain in the lattice. The plot in Fig. 5 illustrates the relationship between β_hkl_cosθ and 4sinθ for the ZnW_12_O_40_/NiZn_2_O_4_. By employing linear regression, the measurements for the size of the crystalline particles (D) and the crystal lattice (ε) were ascertained through the inclination of the line. The lattice strain for (a) NiZn_2_O_4_, (b) ZnW_12_O_4_, and (c) ZnW_12_O_40_/NiZn_2_O_4_ nanocomposite were calculated as − 5.17 $\times$ 10^–3^, 2.42 $\times$ 10^–4^, and 1.6 $\times$ 10^–3^, respectively. The presence of a horizontal line exhibiting both positive and negative slopes can be ascribed to the phenomenon of lattice expansion and compression, respectively. The W–H curve of the ZnW_12_O_40_/NiZn_2_O_4_ nanocomposite exhibits a positive slope, indicating lattice expansion resulting from slight variations in the ionic radius of the constituent elements. Additionally, the size of the crystal in the ZnW_12_O_40_/NiZn_2_O_4_ was found to be about 33 nm, a measurement consistent with the dimensions obtained using the Scherer formula.

#### Morphological observations

**Figure 6:**
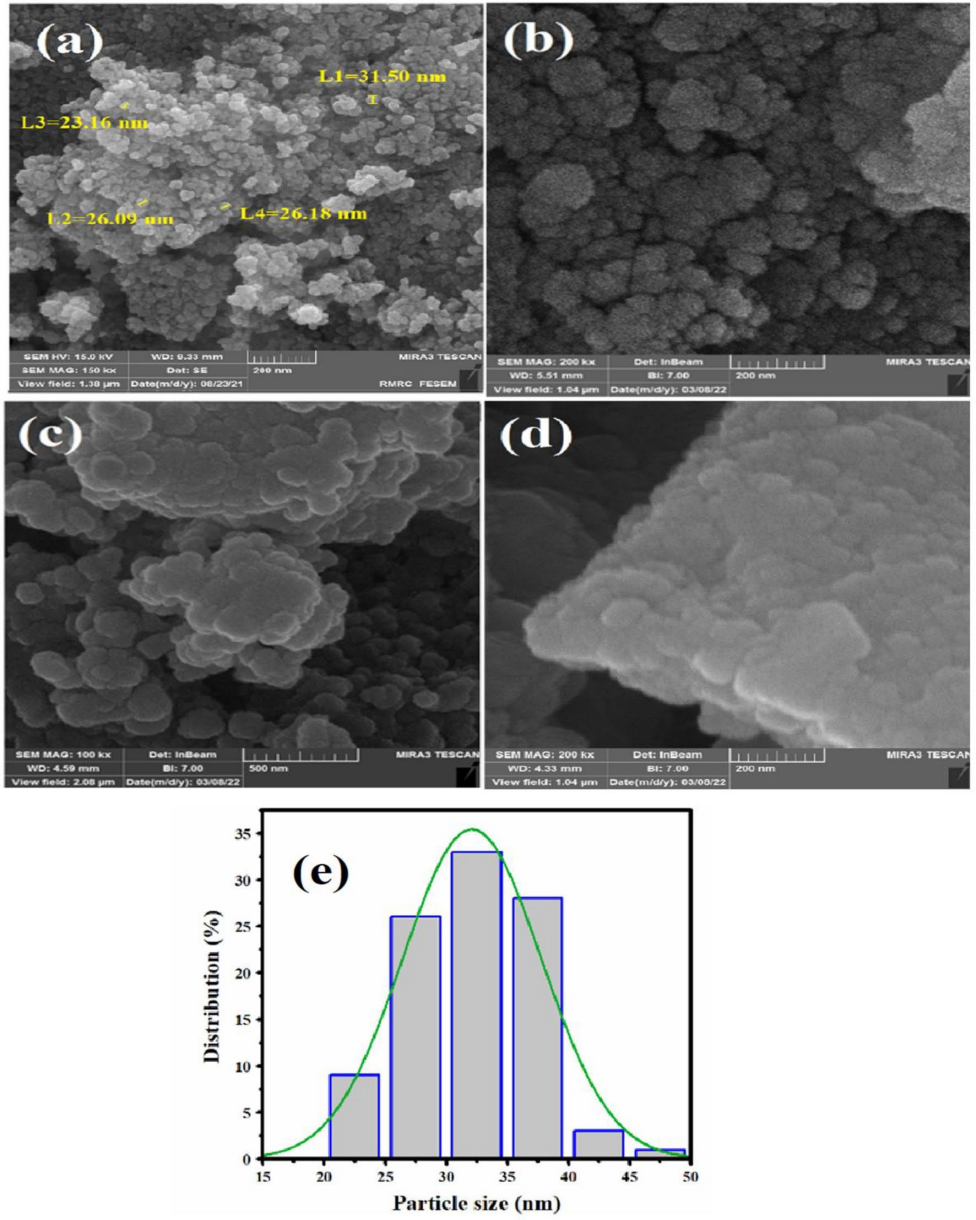
FE-SEM images of (**a**) NiZn_2_O_4_, (**b**) ZnW_12_O_4_, (**c**,**d**) ZnW_12_O_40_/NiZn_2_O_4_, and (**d**) the particle size distribution of the ZnW_12_O_40_/NiZn_2_O_4_ nanoparticles.

The FE-SEM analysis was employed to analyze the surface morphology, estimate the size of the particles, determine the orientation of the particles on the substrate, and identify potential particle accumulation. Figure 6 illustrates the surface morphology obtained from the FE-SEM analysis of (a) NiZn_2_O_4_, (b) ZnW_12_O_4_, and (c) ZnW_12_O_40_/NiZn_2_O_4_ nanocomposite. The arrangement of particles in NiZn_2_O_4_ is observed to be in the form of clumps, resulting in a spherical and relatively uniform shape (Fig. 6a). The surface image of ZnW_12_O_40_–heteropolyoxo revealed irregular accumulations of spherical particles in the nanometer scale (Fig. 6b)^43^. In addition, the FE-SEM image of ZnW_12_O_40_/NiZn_2_O_4_ nanocomposite exhibits that the polyoxometalate is heterogeneously immobilized on NiZn_2_O_4_ with a spherical morphology (Fig. 6c,d). These findings indicate that [ZnW_12_O_40_]^6–^ species was anchored on the surface of NiZn_2_O_4_ nanoparticles. Additionally, the particle size distribution can be analyzed through the histogram depicted in Fig. 6e. Based on the findings, it is evident that the particles are in the range of 30–35 nm. It should be noted that the average size of ZnW_12_O_40_/NiZn_2_O_4_ nanoparticles is about 33 nm.

#### Mapping and EDX micrographs

**Figure 7:**
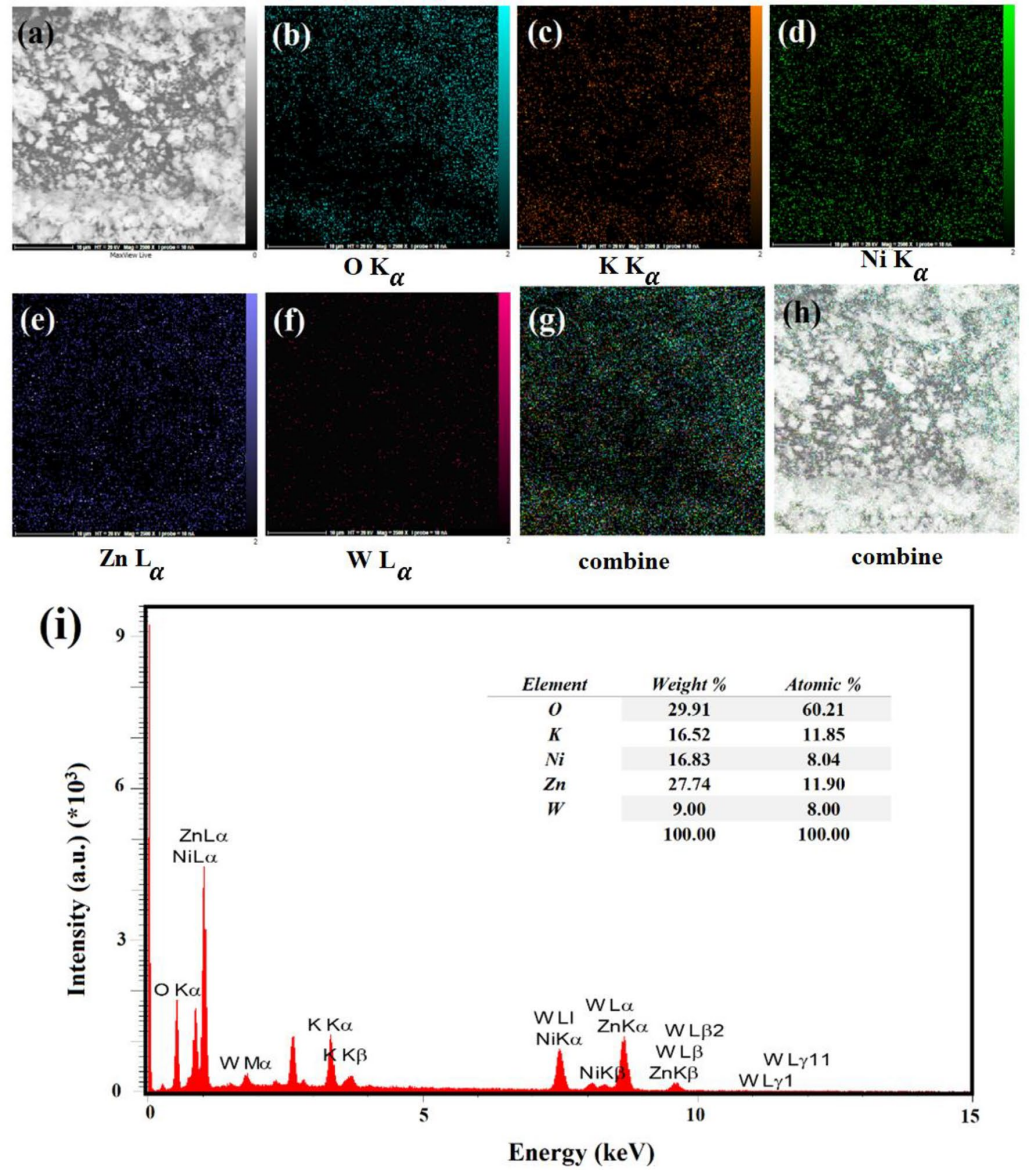
Elemental mapping images (**a**–**h**) and (**i**) EDX micrographs of the ZnW_12_O_40_/NiZn_2_O_4_.

The dispersion of elements was evaluated through Mapping analysis, which was further confirmed by EDX micrographs. The examination conducted by the EDX analysis successfully demonstrates the integration of O, K, W, Ni, and Zn within the structure of the ZnW_12_O_40_/NiZn_2_O_4_ with corresponding weight percentages of 29.91, 16.52, 16.83, 27.74, and 9.00 wt%, respectively (Fig. 7i). As illustrated in Fig. 7a–h, the elemental Mapping of constituents in the ZnW_12_O_40_/NiZn_2_O_4_ aligns with the percentages documented by the EDX analysis. Also, elemental Mapping also displayed that metal oxide nanoparticles were decorated with [ZnW_12_O_40_]^6–^ anionic clusters.

#### Specific surface area and pore size distribution

**Figure 8:**
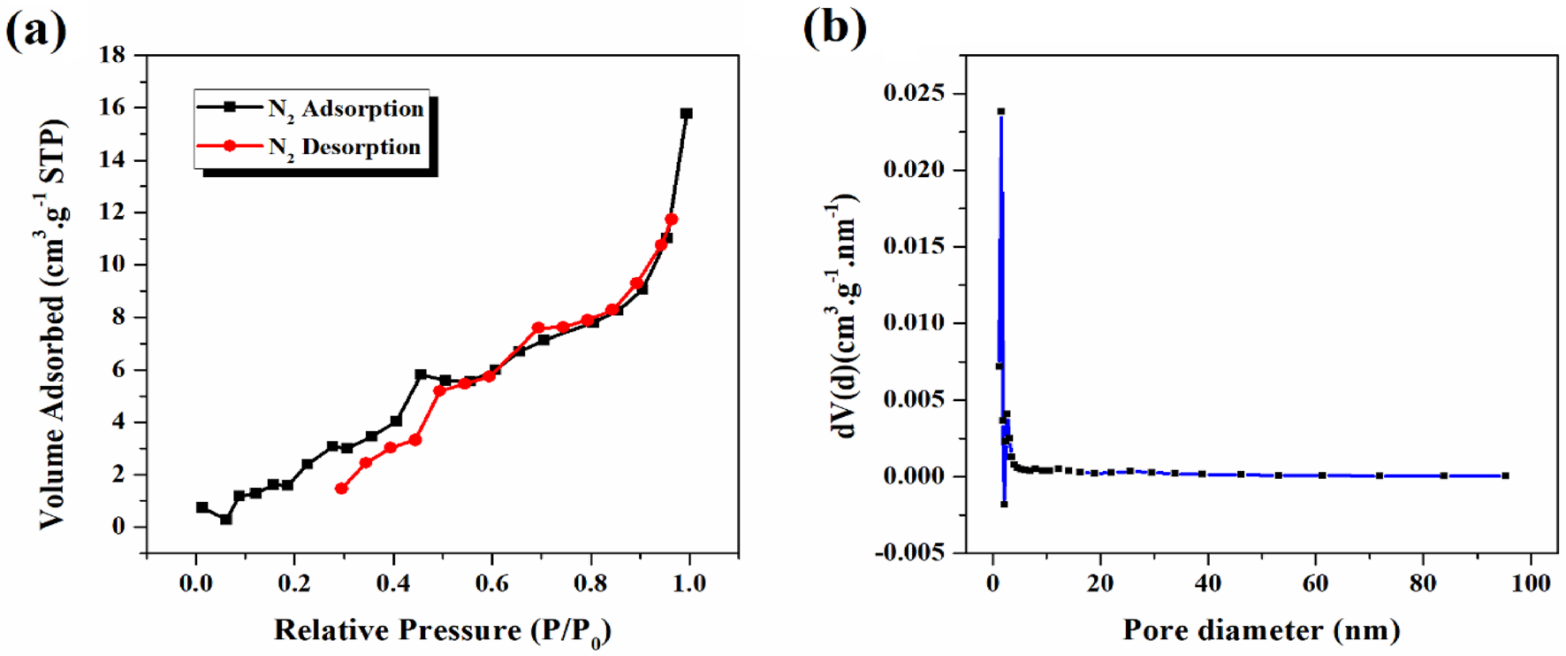
(**a**) Adsorption–desorption isotherm of N_2_ and (**b**) BJH pore size distribution of ZnW_12_O_40_/NiZn_2_O_4_ nanocomposite.

The absorption–desorption isotherm of N_2_ gas and surface characteristics, inclusive of the surface area and pore diameter of the materials were evaluated by the BET and BJH techniques. Figure 8 exhibits the absorption–desorption isotherm of type (II) according to the IUPAC classification, which is characterized by the presence of a microporous or non-porous structure. The specific surface area and pore volume of as-prepared nanocomposite were determined to be 16.572 m^2^ g^−1^ and 0.023584 cm^3^ g^−1^, respectively. Additionally, the findings derived from the BET analysis indicated an average pore diameter of 5.69 nm for the ZnW_12_O_40_/NiZn_2_O_4_ as a potential material for the electrocatalytic hydrogen storage. The specific surface area and porous volume of the ZnW_12_O_40_/NiZn_2_O_4_ are summarized in Table 1.

**Table 1:** Porosity features obtained from BET analysis of ZnW_12_O_40_/NiZn_2_O_4_ nanocomposite.

Sample	BET surface area (m^2^ g^−1^)	Total pore volume (cm^3^ g^−1^)	Average pore diameter (nm)
ZnW_12_O_40_/NiZn_2_O_4_	16.572	0.023584	5.6922

#### TGA-DTG analysis

**Figure 9:**
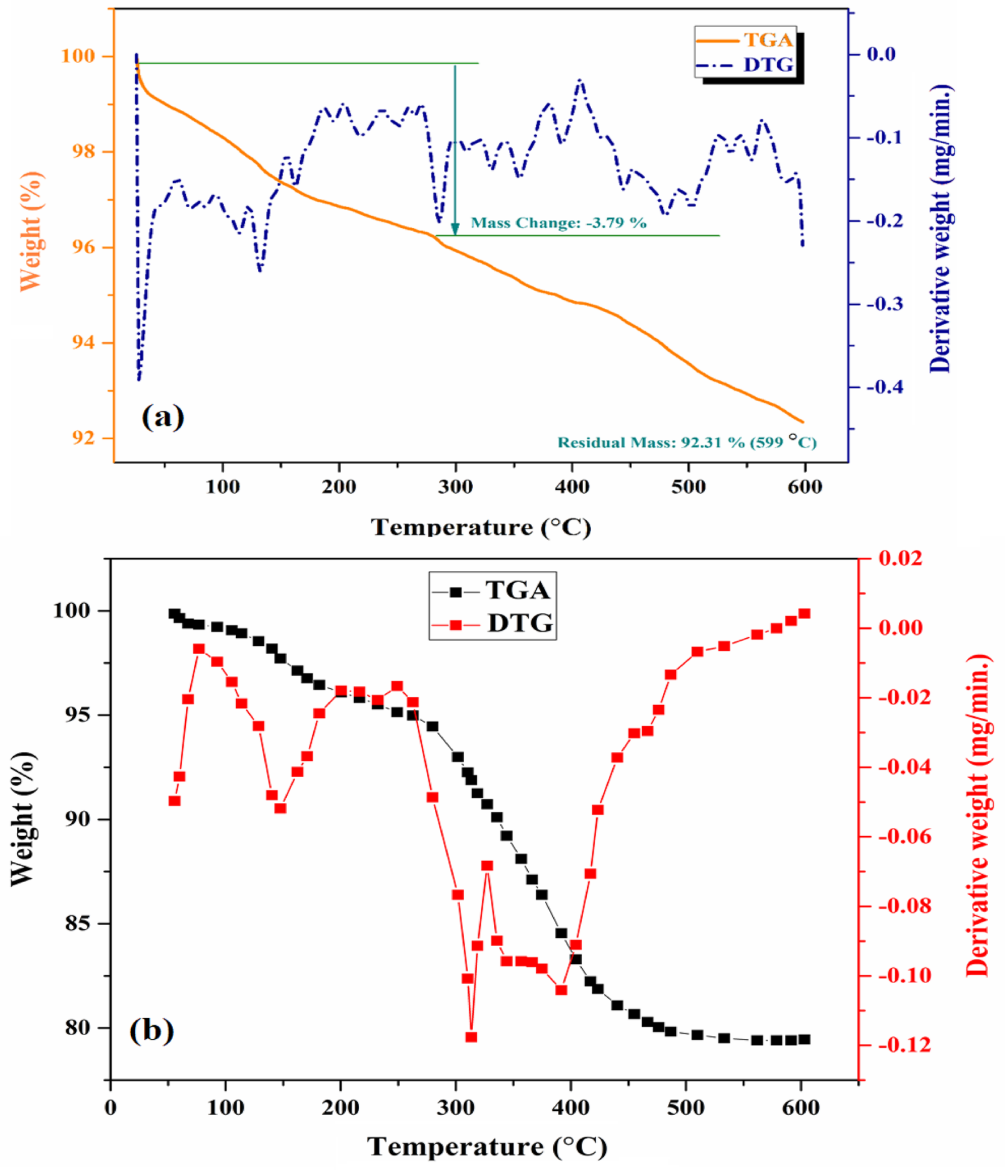
TGA–DTG curve of (**a**) Keggin-type [ZnW_12_O_40_]^6–^ and (**b**) ZnW_12_O_40_/NiZn_2_O_4_ nanocomposite from room temperature to 600 °C.

The TGA-DTG analysis of [ZnW_12_O_40_]^6−^ anionic clusters during pyrolysis in the N_2_ atmosphere is illustrated in Fig. 9. The TGA curve of [ZnW_12_O_40_]^6−^ exhibits several distinct peaks, signifying that Keggin-type ZnW_12_O_40_ experiences several notable weight reduction processes whilst undergoing pyrolysis (Fig. 9a). A significant weight loss (3.90%) was observed at room temperature up to 300 °C, which could be mainly related to the decomposition of adsorbed water molecules. Moreover, a notable weight loss (3.79%) was attributed to the desorption of crystallization water of H_6_[ZnW_12_O_40_]·6H_2_O clusters incorporated into the ZnW_12_O_40_ nanostructure during synthesis. The enhanced mass reduction (92.31%) observed at 559 °C can be ascribed to the withdrawal of oxygen from ZnW_12_O_40_, resulting in its decomposition^44^. Moreover, the TGA-DTG curve for ZnW_12_O_40_/NiZn_2_O_4_ nanocomposite are illustrated in Fig. 9b. The curve shows a two-step decomposition process. The first weight loss (2.79%) at ~ 100 °C is corresponds to the elimination of adsorbed water during the synthesis of as-prepared ZnW_12_O_40_/NiZn_2_O_4_ nanocomposite. The second major weight loss (56.3%) between 250 and 450 °C corresponds to the elimination of capping agent, such as citric acid and urea according to synthesis^45^.

### Electrochemical study results

#### Cyclic voltammetry

**Figure 10:**
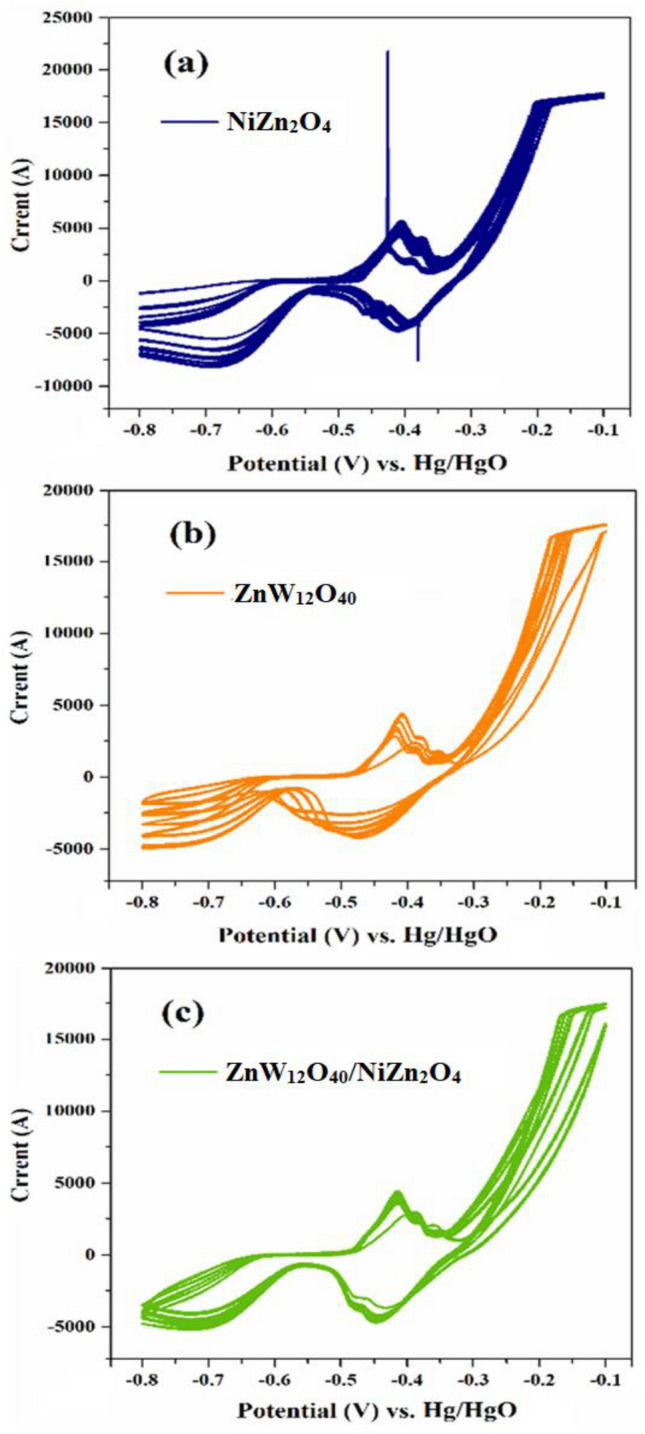
Cyclic voltammetry curves of (**a**) NiZn_2_O_4_, (**b**) ZnW_12_O_40_, and (**c**) ZnW_12_O_40_/NiZn_2_O_4_ as working electrodes.

The electrochemical characteristics of the provided samples were examined by charge–discharge chronopotentiometry (CHP) and cyclic voltammetry (CV) methodologies. Figure 10 illustrates the CV curves of the (a) NiZn_2_O_4_, (b) ZnW_12_O_4_, and (c) ZnW_12_O_40_@NiZn_2_O_4_ in relation to the potential/current of the redox reaction within the range of − 8.0 to − 1.0 V at a constant scan rate of 100.0 mV/s for 20 cycles. As depicted in Fig. 10a, the CV measurements of NiZn_2_O_4_ nanoceramics exhibits the ability to undergo reversible and continuous multi-electron redox reactions on anodic/cathodic sweeps. Furthermore, Keggin-type [ZnW_12_O_40_]^6–^ anionic clusters possess the capability to perform reversible and continuous multi-electron redox, and there are also tungsten ions with different valence states of W^VI^ and W^V^ (Fig. 10b). As seen in Fig. 10c, the redox process of the ZnW_12_O_40_/NiZn_2_O_4_ electrode is under surface control. This phenomenon may be attributed to the presence of Keggin polyanions and the pore structure of the layer.

#### Chronopotentiometry measurements

**Figure 11:**
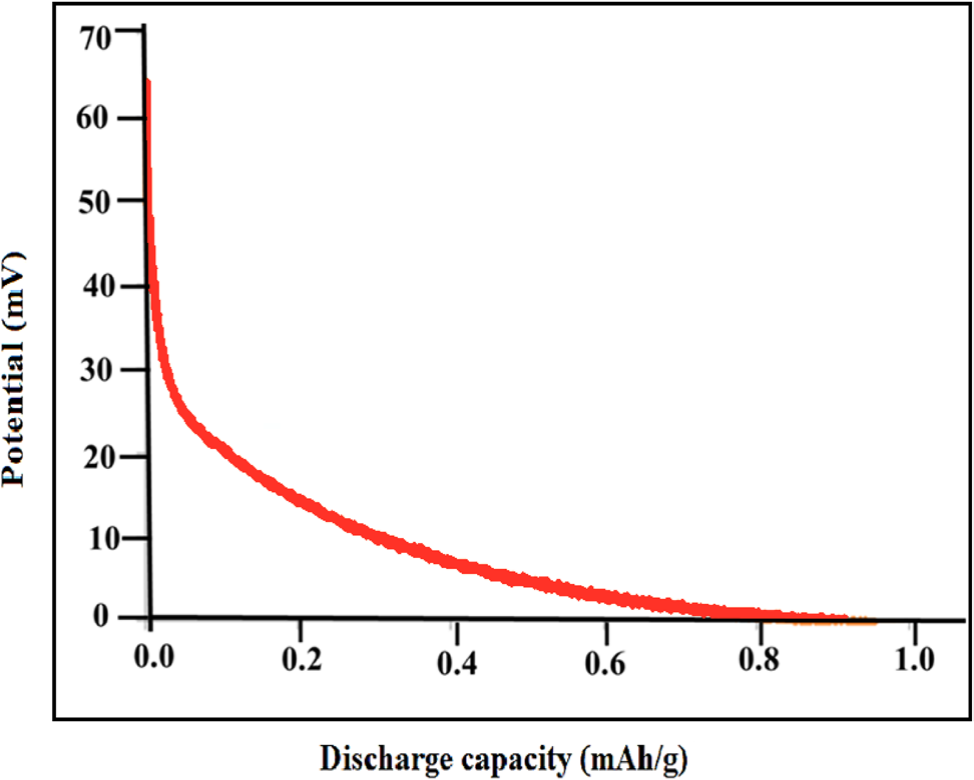
Electrochemical hydrogen storage capacity of copper substrate before coating.

A set of CHP measurements were conducted in order to examine the electrochemical properties of materials. The measurements were carried out with the following specifications: [KOH](aq) = 6.0 M, a scan rate of 100 mV s^−1^, and a three-electrode system comprised of a working counter (Pt) and a reference electrode (Hg/HgO), along with a working electrode formed from the prepared products as active materials. For detailed examination of hydrogen storage capacity, an investigation was conducted on the electrochemical hydrogen storage of bare copper foam, which served as a substrate. It was imperative to present a desorption diagram of the first cycle of the copper foam to demonstrate that it does not affect the discharge capacity of the as-prepared nanocomposite. As indicated in Fig. 11, the discharge capacity of the bare Cu foam revealed a negligible capacity for hydrogen storage ($\sim$ 0.9 mAh/g), which can be disregarded in all stages of the test. As a result, the copper foam can be used as a substrate throughout all electrochemical hydrogen storage procedures.

During the adsorption process, splitting of the water within the electrolyte occurs in immediate proximity to the working electrode. The electrolyte is comprised of H^+^ ions that have the potential to be assimilated by the samples that have been prepared on the working electrode or lead to the reformation and dispersion of hydrogen molecules on the surface of the electrode^46,47^. The resultant reactions can be attributed to the quasi-reversible electrochemical reaction as follows:

3 $$ {\text{Sample }} + {\text{ mH}}_{{2}} {\text{O}} + {\text{me}}^{ - } \leftrightarrow {\text{sample }} + {\text{mH}}^{ + } + {\text{mOH}}^{ - } $$

4 $$ {\text{4OH}}^{ - } \leftrightarrow {\text{O}}_{{2}} + {\text{2H}}_{{2}} {\text{O }} + {\text{4e}}^{ - } $$

In the process of desorption, the release of H + ions occur from the samples and they subsequently react with OH^−^ ions, resulting in the formation of H–O–H molecules. The elucidation of potential reactions taking place during the discharge process can be presented as follows:

5 $$ {\text{Sample }} + {\text{ mH}}^{ + } + {\text{mOH}}^{ - } \leftrightarrow {\text{sample }} + {\text{ mH}}_{{2}} {\text{O }} + {\text{me}}^{ - } $$

6 $$ {\text{O}}_{{2}} + {\text{2H}}_{{2}} {\text{O }} + {\text{4e}}^{ - } \leftrightarrow {\text{4OH}}^{ - } $$

**Figure 12:**
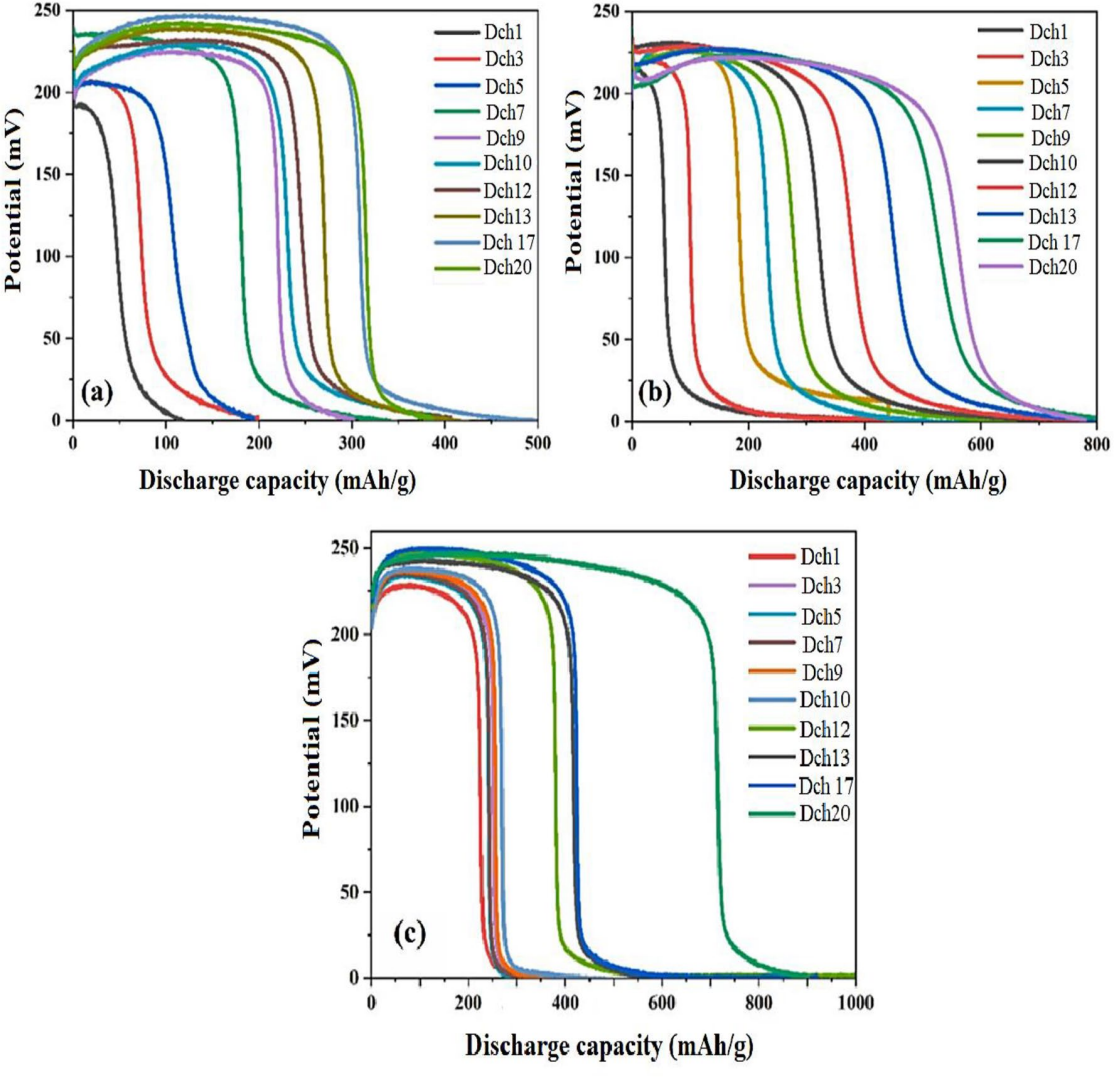
Electrochemical discharge curves of (**a**) NiZn_2_O_4_, (**b**) ZnW_12_O_40_, and (**c**) ZnW_12_O_40_/NiZn_2_O_4_ nanocomposite at a current density of 2 mA.

The hydrogen storage capacity (HSC) of the materials can be calculated using the following equation (Eq. (7)): HSC = It/m, where I represent the applied current (mA), t is the charge–discharge time (h), and m is the amount of the coated nanocomposite (g)^48^. Figure 12a–c illustrates the electrochemical hydrogen storage of (a) NiZn_2_O_4_, (b) ZnW_12_O_4_, and (c) ZnW_12_O_40_@NiZn_2_O_4_ nanocomposite as active materials on working electrodes. After carrying out multiple absorption–desorption cycles at varying currents, a current of 2 mA was identified as the optimal value. The discharge capacity of each cycle demonstrates an increase compared to the preceding cycle when a current of 2 mA is administered. This positive trend persists until the capacity attains its highest achievable value. This enhancement can be attributed to the emergence of new sites that facilitate the absorption–desorption of hydrogen. In the applied of current of 2 mA, the discharge capacity of the NiZn_2_O_4_ nanoparticles experienced a significant increase from approximately 110 to 420 mAh/g after 20 cycles (Fig. 12a). Furthermore, the Keggin-type [ZnW_12_O_40_]^6^ cluster exhibited a notable discharge capacity ($\sim$ 360 mAh/g) during the initial cycle, and the storage capability progressively amplified to about 790 mAh/g after 20 cycles (Fig. 12b). As seen in Fig. 12c, the discharge capacity of the ZnW_12_O_40_@NiZn_2_O_4_ nanocomposite has demonstrated a significant enhancement from 340 to 900 mAh/g under identical experimental conditions after 20 runs. The data presented in this research demonstrates a positive correlation between the surface area and the electrochemical hydrogen storage capacity. As a result, the ZnW_12_O_40_@NiZn_2_O_4_ nanocomposite proves to be a preferred active material for hydrogen adsorption and desorption due to its larger surface area, allowing for a stronger interaction with H_2_ molecules. Further, it can be inferred that the remarkable efficacy of electrochemical hydrogen storage can be ascribed to the synergistic influence of ZnW_12_O_40_ and NiZn_2_O_4_ nanostructures.

The discharge capacity of several materials was documented in Table 2 alongside their corresponding formula structures. Among the diverse nanoparticles and composites, the optimum efficiency was associated with ZnW_12_O_40_/NiZn_2_O_4_ nanocomposite under experimental condition.

**Table 2:** Comparison of electrochemical hydrogen storage between ZnW_12_O_40_/NiZn_2_O_4_ and other nanomaterials.

Material	KOH concentration	Number of cycles	Discharge capacity (mAh/g)	References
SnFe_2_O_4_	6 M	20	460	^39^
DyFeO3-ZnO	2 M	15	600.11	^49^
La_2_Ti_2_O_7_	6 M	50	224	^50^
Ce_2_W_2_O_9_/CoWO_4_/PC^a^	2 M	15	660	^51^
TiO_2_- decorated MWCNT	7 M	23	540	^52^
NiZn_2_O_4_	6 M	20	420	This study^b^
ZnW_12_O_40_	6 M	20	790	This study^b^
ZnW_12_O_40_/NiZn_2_O_4_	6 M	20	900	This study^b^

^a^Porous carbon.

^b^Condition of experiment: [KOH] (aq) = 6.0 M, a scan rate of 100 mV s^−1^, current density of 2 mA, and a three-electrode system comprised of a working counter (Pt) and a reference electrode (Hg/HgO), along with a working electrode formed from the prepared products.

## Conclusions

In the current study, a new nanocomposite was synthesized successfully through the incorporation of [ZnW_12_O_40_]^6–^ anionic clusters onto NiZn_2_O_4_ particles and applied in the electrochemical hydrogen storage process. The as-prepared nanocomposite indicates a spherical morphology with an average diameter of approximately 32.9 nm, which was calculated by the Scherer equation. Different parameters, such as discharge capacity of the bare copper foam, and the influence of repeated hydrogen adsorption–desorption cycles were discussed. The electrochemical findings revealed that the ZnW_12_O_40_/NiZn_2_O_4_ nanocomposite exhibits a remarkable capacity and outstanding reversibility in comparison to individual ZnW_12_O_40_ and NiZn_2_O_4_ nanoparticles for the purpose of hydrogen storage. The highest hydrogen discharge capability of the ZnW_12_O_40_/NiZn_2_O_4_ was achieved $\sim$ 340 mAh/g during the 1st cycle, and the storage capacity enhanced to approximately 900 mAh/g at the end of 20 cycles using a current density of 2 mA. Hence, the ZnW_12_O_40_/NiZn_2_O_4_ nanocomposite can effectively contribute to the field of energy storage as a promising and innovative candidate material.

## Data Availability

Data will available when requested from outers or from corresponding author.
